# Case Report: Adult Post-COVID-19 Multisystem Inflammatory Syndrome and Thrombotic Microangiopathy

**DOI:** 10.3389/fimmu.2021.680567

**Published:** 2021-06-23

**Authors:** Idris Boudhabhay, Marion Rabant, Lubka T. Roumenina, Louis-Marie Coupry, Victoria Poillerat, Armance Marchal, Véronique Frémeaux-Bacchi, Khalil El Karoui, Mehran Monchi, Franck Pourcine

**Affiliations:** ^1^ Groupe Hospitalier Sud Ile de France, Service de Réanimation, Melun, France; ^2^ Centre Hospitalo-Universitaire Necker, Service d’Anatomie Pathologique, Paris, France; ^3^ Centre de Recherche des Cordeliers, INSERM, Sorbonne Université, Université de Paris, Paris, France; ^4^ Hôpital Européen Georges Pompidou, Laboratoire d’Immunologie Biologique, Paris, France; ^5^ Centre Hospitalo-Universitaire Henri Mondor, Service de Néphrologie et Transplantation, Créteil, France; ^6^ Groupe Hospitalier Sud Ile de France, Service de Néphrologie, Melun, France

**Keywords:** thrombotic microangiopathy, multisystem inflammatory syndrome, COVID-19, complement system, eculizumab, case report

## Abstract

**Background:**

The coronavirus disease 2019 (COVID-19) pandemic has affected millions of people worldwide. A clinical series of Kawasaki-like multisystem inflammatory syndrome (MIS), occurring after SARS-CoV-2 infection, have been described in children (MIS-C) and adults (MIS-A), but the pathophysiology remains unknown.

**Case Presentation:**

We describe a case of post-COVID-19 MIS-A in a 46-year-old man with biopsy-proven renal thrombotic microangiopathy (TMA). Specific complement inhibition with eculizumab was initiated promptly and led to a dramatic improvement of renal function.

**Conclusion:**

Our case suggests that that TMA could play a central role in the pathophysiology of post-COVID-19 MIS-A, making complement blockers an interesting therapeutic option.

## Introduction

The coronavirus disease 2019 (COVID-19) pandemic caused by SARS-CoV-2 has affected millions of people worldwide. In adults, COVID-19 is typically characterised by severe interstitial pneumonia and hyperactivation of the inflammatory cascade (1). There is growing evidence that COVID-19 affects the endothelial system, leading to endothelial dysfunction characterised by a pro-inflammatory and pro-coagulative state (2–5). Clinical series of Kawasaki-like multisystem inflammatory syndrome (MIS), occurring after viral clearance, have been described in children (MIS-C) (6–9). Recently, similar case series of MIS were described in adults (MIS-A) (10–15). However, the pathophysiology of MIS remains unknown. We report a case of MIS-A with biopsy-proven thrombotic microangiopathy (TMA) successfully treated with eculizumab.

## Case Presentation

**Figure 1:**
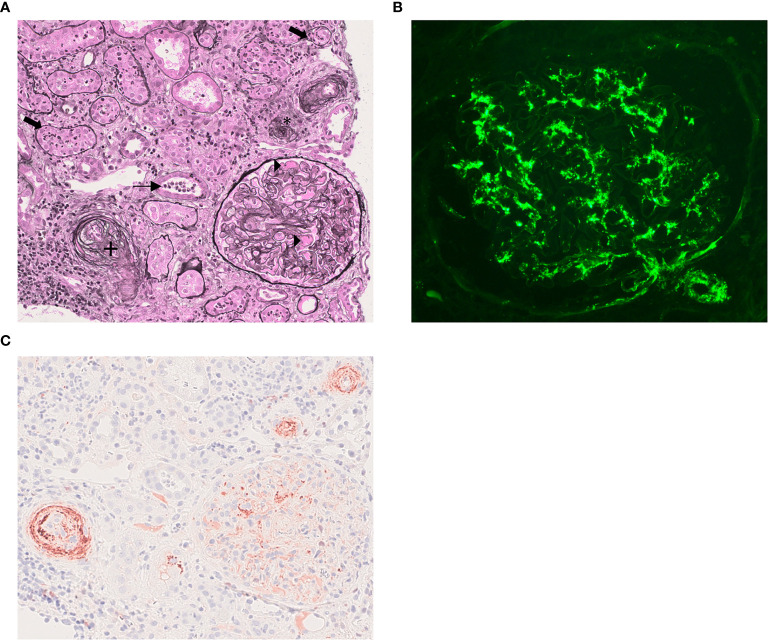
Kidney biopsy findings. **(A)** Light microscopy analysis: Fibrin within glomerular capillary loops (black triangle). Small inter-lobular artery with intimal mucoid alterations and endothelial cells swelling (+). Arteriolar occlusion (*). Polymorphonuclear infiltration with tubulitis (thick arrows) and granular casts (thin arrow) (Jones methanamine silver staining, x200). **(B)** Immunofluorescence stucy showing predominant mesangial and sub-endothelial C3 deposits in glumeruli and in glomerular arteriole. (Anti-C3c fluorescein isothiocyanate-conjugate x400). **(C)** Immunochemistry study showing positive C5b-9 staining in arterioles, inter-lobular artery and glomeruli within the mesangium (Mouse IgG, B7 antineoepitope, ^1^x40). ^1^Kindly provided by Prof. P. Morgan (Cardiff Intitute of Infection&Immunity, UK).

A 46-year-old patient of West African ancestry was admitted to our hospital for hypertensive emergency (189/123 mmHg) and fever. He had a personal history of arterial hypertension and obesity (BMI = 36 kg/m^2^) and family history of arterial hypertension. No previous COVID-19 symptoms were reported, and the patient did not take any prescribed or over-the-counter medications. Physical examination was normal. SARS-CoV-2 PCR of nasopharyngeal swab was negative (repeated twice), but COVID-19 serology was positive for IgG (80 UA/mL, positive if > 12 UA/mL, Immunoassay YHLO iFlash 1800) and negative for IgM. Thoracoabdominopelvic CT scan was unremarkable. First investigations revealed an inflammatory state, anaemia, thrombocytopenia and acute kidney injury (AKI). The serum creatinine (sCr) level was 169 µmol/L and associated with 1g per day proteinuria, aseptic pyuria, no haematuria and low natriuresis (< 20 mmol/L). C-reactive protein (CRP) level was 312 mg/L and neutrophil count was 18.7 g/L (**Table 1**). On day 4, the patient presented with evanescent facial erythema and developed acute myocardial dysfunction with reduced left ventricular ejection fraction (40%), pericardial effusion and elevation of high-sensitivity troponin (hsTroponin). Taking into account the frequency of vascular thromboses related to COVID-19, therapeutic anticoagulation with heparin was started. On day 5, neurological impairment appeared with coma, leading to intubation and mechanical ventilation. Cerebrospinal fluid analysis was unremarkable. Abnormal supratentorial periventricular MRI signals responsible for a restriction of the diffusion testified to acute vasculitis. No immunosuppressive treatment was introduced because of concomitant tracheal aspiration positive for *Enterobacter aerogenes*, which was treated with trimethoprim-sulfamethoxazole. On day 7, myocardial and renal function worsened (sCr 660 µmol/L), requiring initiation of dobutamine and intermittent renal replacement therapy (RRT). A kidney biopsy was performed. Light microscopy revealed typical lesions of TMA, including fibrin thrombi within glomeruli and myxoid intimal alterations of arterioles and small-to-medium sized renal arteries. The remaining glomeruli were normal without hypercellularity. A significant interstitial infiltrate, mainly composed of neutrophils, was responsible for severe tubulitis and moderate acute tubular necrosis (**Figure 1A**). Immunofluorescence study showed isolated mesangial complement C3c-positive deposits without evidence for IgG, IgA, IgM, C1q or C4d deposits (**Figure 1B**). Immunochemistry study showed C5b-9 deposits at the same localisation (**Figure 1C**). Immunological work-up is shown in **Table 2**. ADAMTS13 activity was moderately decreased but did not reach the cut-off for a diagnosis of thrombotic thrombocytopenic purpura. Complement work-up evaluation found an elevated soluble C5b-9 (sC5b-9) with low C4 and normal C3 levels in the serum (**Table 2**). Cryoglobulinemia was negative. All coding sequences of *CFH*, *CFI, MCP, C3, CFB* and *THBD *genes were analysed by next-generation sequencing. We defined a variant as rare when its minor allele frequency was below 1% in the general population. No rare variants were detected in the six complement genes implicated in atypical haemolytic uremic syndrome (aHUS).

**Table 1:** Clinical and laboratory findings.

		****	Days from hospital admission																			
**Finding**		**1**	**2**	**3**	**4**	**5**	**6**	**7**	8*	**9**	**10**	**11**	**12**	**13**	**14**	15*	**16**	**17**	**18**	**19**	**30**	**182**
**Clinical**																						
**Respiratory status**		SB	SB	SB	O2	MV	MV	MV	MV	MV	MV	MV	MV	SB	SB	SB	SB	SB	SB	SB	SB	SB
**Dobutamine (gamma/kg/min)**		–	–	–	–	–	–	5.75	10	10	10	5	–	–	–	–	–	–	–	–	–	–
**Urine output (L/day)**		–	–	–	0.3	0.3	0.5	0.7	0.8	0.9	3	7.8	6.9	7.4	5.2	6.3	4.2	2.8	2.7	2.2	1.5	–
**Temperature (°C)**		40.7	40.6	40	40.2	39.1	38.7	38.4	37.3	38.1	36.3	36.8	36.2	36.1	36.2	36	35.7	34.7	35.7	36	36	37
**Laboratory**	**Normal values**																					
**Creatinine (µmol/L)**	[59-104]	169	225	348	536	627	666	RRT	691	RRT	441	395	324	256	208	167	147	130	151	129	109	82
**CRP (mg/L)**	[<5]	312	469	528	621	551	462	319	292	213	153	87	58	45	32.9	25	19	12.9	11.4	8.8	3	3.7
**Leucocyte count (G/L)**	[4-10]	18.7	15.7	20	25	25.1	26.8	25.7	26.7	19.9	17	12.3	11.9	10.3	9.3	7.7	8	5.5	4.4	3.3	5.7	8.6
**Haemoglobin level (g/dL)**	[13-16.7]	12.3	9.8	8.3	7.5	7.1	6.6	7.3	7	6.2	6.7	7.6	7.8	8.7	8.7	9.2	9.7	8.8	9.7	9.3	12.6	13.3
**Platelet count (G/L)**	[150-450]	98	90	97	288	392	450	392	339	260	261	267	267	311	315	330	347	304	326	300	217	389
**LDH (UI/L)**	[135-225]	461	432	473	–	–	–	491	636	435	449	413	386	–	372	358	333	280	288	243	–	–
**hsTroponin (ng/L)**	[<14]	25	74	–	1006	1700	2791	2464	1669	–	1048	628	481	294	214	130	98.7	–	120	140	28	21.6
**Haptoglobin (g/L)**	[0.3-2]	–	–	–	2.91	2.91	–	–	–	–	–	–	–	–	–	–	–	2.67	2.41	2.71	–	–
**Ferritin (µg/L)**	[30-400]	–	3206	–	–	3538	–	–	–	–	–	–	–	–	–	–	–	–	–	–	–	–
**Schistocytes (%)**	<1%	–	–	< 1	<1	–	–	–	–	–	–	–	–	–	–	–	–	–	–	–	<1	–
**UPCR (mg/g)**	<300	996	–	–	–	–	686	–	–	–	–	–	–	–	–	–	–	–	–	–	90	90
**Others**	HBV, HCV and HIV 1 serologies: negatives; glycosylated ferritin 12% (N > 20%);																					

HBV, hepatitis B virus; HCV, hepatitis C virus; HIV, human immunodeficiency Virus; hsTroponin, High-sensitive Troponin; LDH, Lactate dehydrogenase; MV, mechanical ventilation; O2, oxygen therapy; RRT, renal replacement therapy; SB, spontaneous breathing; UPCR, urine protein creatinine ratio.

*eculizumab 900mg.

Bold data on the first line correspond to the days from patient’s hospital admission.

**Table 2:** Immunological work-up.

Laboratory	Normal values	Day 1	Day 182
**ADAMTS-13 activity (%)**	50-150	26	–
**CH 50 (%)**	70-130	64	106
**C3 (mg/L)**	660-1250	1030	1360
**C4 (mg/L)**	93-380	69	314
**C1q (mg/L)**	125-225	227	–
**Factor H (%)**	65-140	93	116
**Factor I (%)**	70-130	122	134
**sC5b9 (ng/mL)**	<420	469	–
**Properdin (mg/L)**	12-40	38	–
**C1 inhibitor antigen (mg/L)**	170-540	357	–
**C1 inhibitor function (%)**	70-130	>125	–
**Anti-Factor H antibodies**	–	negative	negative
**Antinuclear antibodies**	–	1/160	–
**Anti-DNA antibodies**	–	negative	–
**Cryoglobulinemia**	–	negative	–
**Anti-cardiolipin antibodies**	–	negative	–
**Anti-B2GP1 antibodies**	–	negative	–
**Lupus anticoagulant**	–	negative	–

ADAMTS13, a disintegrin and metalloprotease with thrombospondin type I repeats-13; sC5b9, soluble C5b-9 plasmatic level; DNA, desoxyribonucleic acid; B2GP1, beta 2 glycoprotein 1.

On day 8, specific complement inhibition with eculizumab (900 mg) was initiated. Three days later, cardiac function and neurological impairment improved, urine output increased, and blood creatinine decreased, allowing the withdrawal of dobutamine, RRT and mechanical ventilation (**Table 1**). On day 15, the patient received a second and last dose of eculizumab (900 mg). On day 30, the patient was discharged from the hospital, with a sCr 109 µmol/L and cardiac MRI showing no pericardial effusion, no sequelae of segmental hypokinesia and a left ventricular ejection fraction of 50%. Six months later, the patient resumed normal activities of daily living. Left ventricular function has normalised, despite persistent arterial hypertension. sCr is 82 µmol/L, without significant albuminuria (**Tables 1** and **2**).

## Discussion and Conclusion

We describe the first case of post-COVID19 MIS-A associated with renal TMA successfully treated with eculizumab.

In this case, the IgG-positive serology, negative PCR swab and the absence of pulmonary involvement demonstrate the post-infectious nature of this syndrome, occurring after viral clearance.

Kidney involvement is frequent in COVID-19, as more than 40% of cases have abnormal proteinuria at hospital admission (16, 17). Scarce histological data are available, showing in most cases ATN, collapsing glomerulopathy or TMA in patients with acute COVID-19 infection (18–22). AKI is also common during MIS-C, ranging from 10% to 60% of the cases (6, 23, 24) while it was described in four adults in a case series of 20 MIS-A with cardiac involvement (12). Currently, the pathogenesis of AKI in MIS is thought to be mainly related to cytokine-mediated hypotension and cardiac dysfunction, leading to renal hypoperfusion (25).

Our case describes the first kidney biopsy performed in a patient with MIS-A. Scarce histological data are available on this syndrome. A first report showed intraepithelial collections of neutrophils with necrotic keratinocytes in skin biopsy (10). Likewise, in a fatal case of MIS-A, cardiac vasculitis composed of numerous neutrophils and CD4+ T cells was described (14). Similarly, in our case, renal biopsy revealed an aggressive interstitial infiltrate, mainly composed of neutrophils together with TMA.

TMA refers to pathological features of microvascular injury, including thrombi of platelets and fibrin in capillaries and arterioles (26, 27). These lesions are usually associated with peripheral thrombocytopenia and mechanical haemolytic anaemia, although some of these biological markers may be absent (27, 28). In our patient, the absence of a decreased haptoglobin level could be explained by the intensity of the inflammatory syndrome and predominant intrarenal TMA. Diorio et al. recently proposed criteria for clinical TMA associated with MIS-C, including: schistocytes on blood smear, anaemia, elevated LDH, new thrombocytopenia, anaemia, proteinuria, hypertension and elevated sC5b9 (29). Our patient fulfilled five out of seven of the criteria, thus meeting their definition. aHUS is a form of TMA with predominant kidney involvement. The pathophysiology of aHUS involves multiple hits (30), but complement activation has a crucial role in this syndrome. Genetically determined or acquired dysregulation of the complement alternative pathway (CAP) has been found in up to 70% of patients with aHUS (27). Genetic screening was negative in our patient. Although we cannot exclude an unknown variant, it is likely that our patient presented with MIS-A complicated with TMA, rather than aHUS unmasked by SARS-CoV-2 infection.

The complement system (CS) seems to play a pivotal role in the pathophysiology of COVID-19, as few series have reported TMA injury in lungs and skin with sustained activation of CAP and lectin pathway during COVID-19 disease (31–33). Moreover, mice lacking complement component C3, display less severe respiratory failure and inflammatory syndrome after *SARS-Cov* infection (34). Likewise, complement overactivation likely contributes to the renal injury during the course of COVID-19 infection, since a few studies have showed complement deposits in vascular beds and tubules (35). In this case, low serum C4 with normal C3 and mildly elevated sC5b-9 is suggestive of classical and/or lectin complement pathways activation. As MIS-A is a post-infectious immune-mediated phenomenon, anti-SARS-CoV-2 immune complexes could drive complement activation. However, Diorio et al. found no correlation between SARS-CoV-2 antibodies and sC5b-9 elevations (29). Moreover, in our case, histopathological analysis revealed evidence of TMA together with C3c deposits but without C4d or immunoglobulin deposits, which suggestive of alternative complement pathway activation. Lectin pathway triggering, though, cannot be excluded, since it can occur in a C4-bypass pathway and MASP-2 has been suggested to play a key role in the disease process of COVID-19 (33, 36, 37). In our patient, sC5b-9 levels were elevated and C5b-9 staining was positive in kidney biopsy, which is indicative of C5 cleavage by C5 convertase, as described in patients with COVID-19 (38). Likewise, Diorio et al. studied 50 hospitalised paediatric patients with acute SARS-CoV-2 infection (n=21 minimal COVID-19; n=11 severe COVID-19 and n=18 MIS-C) (29); 11 of 18 patients with MIS-C met clinical criteria for TMA. The median sC5b-9 was higher in the patients meeting TMA criteria and associated with AKI. None of the 18 patients needed RRT and no kidney biopsy was performed. Noteworthy, sC5b-9 was also elevated in patients with minimal COVID-19 disease. Eculizumab is a monoclonal anti-C5 antibody that blocks the formation of the membrane attack complex on the surface of endothelial cells and has revolutionised the prognosis of aHUS (39, 40). Small case series have suggested the potential benefits of eculizumab in COVID-19 (41–43). However, no randomized clinical trial has been published to date (32). In our patient, kidney function improved after eculizumab. However, fever, thrombocytopenia and troponin levels were already improving before using any complement blockade. Six months later and after only two courses of eculizumab, our patient’s kidney function has normalised without albuminuria. Likewise, in the case series of MIS-C with TMA published by Diorio et al., all the children recovered (29). Therefore, we cannot exclude that improvement was due to the natural course of the disease rather than to eculizumab, as described in HUS caused by an infection from shiga toxin-producing Escherichia coli (STEC) (27). Complement blockers have never been tested in patients with MIS, except in a 14-year-old child with features of both acute COVID-19 infection and MIS-C, who developed TMA (44). In this case, a kidney biopsy could not be performed, but AKI resolved on eculizumab, as in our patient.

In conclusion, our case suggests that TMA could play a central role in the pathophysiology of post COVID-19 MIS, making complement blockers an interesting therapeutic option.

## Data Availability Statement

The original contributions presented in the study are included in the article/supplementary material. Further inquiries can be directed to the corresponding author.

## Ethics Statement

Written informed consent was obtained from the individual(s) for the publication of any potentially identifiable images or data included in this article.

## Author Contributions

IB and FP wrote the manuscript. MR is the pathologist who made the diagnosis and took the photographs in **Figure 1**. KE-K, L-MC, LR, and MM participated in proofreading and collection of data. AM performed the biochemical analysis of the complement pathway. VP performed C5b-9 staining on the kidney biopsy. VF-B performed genetic analysis. FP is the corresponding author. All authors contributed to the article and approved the submitted version.

## Conflict of Interest

The authors declare that the research was conducted in the absence of any commercial or financial relationships that could be construed as a potential conflict of interest.

## Abbreviations

aHUS, atypical haemolytic uremic syndrome; AKI, acute kidney injury; BMI, body mass index; CFB, Complement factor B; CFH, Complement factor H; CFI, Complement factor I; MCP, Membrane Cofactor Protein; CRP, C-reactive protein; hsTroponin, high-sensitivity troponin; MAC, membrane attack complex; MIS-C, multisystem inflammatory syndrome in children; MIS-A, multisystem inflammatory syndrome in adults; PCR, polymerase chain reaction; RRT, renal replacement therapy; sCr, serum creatinine; sC5b-9, soluble C5b-9; TMA, thrombotic microangiopathy; THBD, Thrombomodulin.
